# Scalable bottom-up synthesis of Co-Ni–doped graphene

**DOI:** 10.1126/sciadv.ado8956

**Published:** 2024-11-08

**Authors:** Valeria Chesnyak, Daniele Perilli, Mirco Panighel, Alessandro Namar, Alexander Markevich, Thuy An Bui, Aldo Ugolotti, Ayesha Farooq, Matus Stredansky, Clara Kofler, Cinzia Cepek, Giovanni Comelli, Jani Kotakoski, Cristiana Di Valentin, Cristina Africh

**Affiliations:** ^1^ Physics Department, University of Trieste, via A. Valerio 2, Trieste 34127, Italy.; ^2^ CNR-Istituto Officina dei Materiali (IOM), Strada Statale 14, km 163.5, 34149 Trieste, Italy.; ^3^ Dipartimento di Scienza dei Materiali, Università di Milano-Bicocca, via R. Cozzi 55, I-20125 Milano, Italy.; ^4^ University of Vienna, Faculty of Physics, Boltzmanngasse 5, 1090 Vienna, Austria.

## Abstract

Introducing heteroatoms into graphene is a powerful strategy to modulate its catalytic, electronic, and magnetic properties. At variance with the cases of nitrogen (N)– and boron (B)–doped graphene, a scalable method for incorporating transition metal atoms in the carbon (C) mesh is currently lacking, limiting the applicative interest of model system studies. This work presents a during-growth synthesis enabling the incorporation of cobalt (Co) alongside nickel (Ni) atoms in graphene on a Ni(111) substrate. Single atoms are covalently stabilized within graphene double vacancies, with a Co load ranging from 0.07 to 0.22% relative to C atoms, controllable by synthesis parameters. Structural characterization involves variable-temperature scanning tunneling microscopy and ab initio calculations. The Co- and Ni-codoped layer is transferred onto a transmission electron microscopy grid, confirming stability through scanning transmission electron microscopy and electron energy loss spectroscopy. This method holds promise for applications in spintronics, gas sensing, electrochemistry and catalysis, and potential extension to graphene incorporation of similar metals.

## Teaser

Single Co and Ni atoms are incorporated in electrochemically detachable graphene layers with a one-step method.

## INTRODUCTION

Since 2004, when graphene (Gr) was first isolated, it has attracted the interest of researchers worldwide due to its exceptional mechanical and physicochemical properties. However, despite the extensive efforts to harness its remarkable attributes and tailor them for specific applications, a definitive flagship application for Gr has yet to emerge. The introduction of substitutional heteroatoms in Gr has been investigated for many years as a strategy to tune its properties (*1*, *2*). The plethora of important applications of such doped Gr layers, with high scientific and technological impact, includes catalysis and electrochemistry (*3*–*9*), energy storage and conversion (*10*), gas sensing (*11*, *12*), spintronics (*13*, *14*), electronics and optoelectronics (*15*), and even biomedics (*16*).

In this context, it is predicted that Co single atoms incorporated into or adsorbed onto Gr exhibit distinctive catalytic activity (*17*) and magnetic (*18*, *19*) properties. While experimental investigations of B- and/or N-doped Gr have flourished in recent years (*20*), reports concerning transition metal (TM)–doped layers are less common due to the lack of a scalable synthesis method (*21*) similar to those reported for B and N, which include simple during-growth processes (*22*, *23*). On the other hand, Gr doping by metal atoms is typically achieved through coordination with four pyridinic N atoms in a porphyrin-like structure (*4*, *6*, *24*) and not by directly embedding the single metal atom through covalent C─TM bonds. As a consequence, the incorporation of TM atoms in substantial concentrations via direct metal-to-Gr (M─Gr) bonding remains a challenging task. The inclusion of substitutional Au (*25*), Al (*26*), Fe (*27*), Mn (*28*), Pd and Pt (*24*), Ni (*29*), and other metal atoms (*30*) in Gr was recently demonstrated, but the underlying implantation methods, such as beam dragging insertion (*30*), require complicated processes and inefficient resource usage, resulting in rather low concentrations and locally constrained distributions. More uniform dopant abundance is achieved by ultralow-energy ion implantation (*31*) or two-step ion implantation (*25*, *32*); yet, these top-down approaches include post-growth defect formation and lead to an overall deterioration of the Gr quality, as not all defects host the desired dopant. So far, the best doping strategies yielding high-quality Gr, high dopant concentrations, and uniform distribution are those that take place through during-growth processes, e.g., using molecular precursors to dope the Gr layer with B (*22*). However, methods that work for low solubility elements, such as B and N, do not apply to TMs, which typically dissolve into the substrate bulk at the temperatures required to grow Gr, resulting in the formation of alloys (*33*).

On Ni substrates, Gr grows via chemical vapor deposition (CVD) at rather low temperatures, starting from 350°C (*34*). Typically, the overall time required to form a complete monolayer (ML) on Ni(111) substrates, at typical hydrocarbon pressure exposures (5 × 10^−8^ to 1 × 10^−6^ mbar) and Gr formation temperatures, is around 1 hour. This means that the growth dynamics are rather slow, allowing Ni atoms to diffuse across the surface until they interact with the growing Gr edge (*35*), remaining sometimes trapped inside the C-network, almost exclusively in double vacancies (DVs) (*29*). The resulting layer, referred to as Ni-doped Gr in the following, is therefore a Gr network, epitaxial on Ni(111), where single Ni atoms are trapped in the C-network. The unique combination of low temperature and slow kinetics for the Gr growth on Ni substrates is the basis of this study, where we synthesized Co- and Ni-codoped Gr layers (Co-Ni–doped Gr) and characterized them by variable-temperature scanning tunneling microscopy (VT-STM) accompanied by density functional theory (DFT) calculations. The subtle different appearance of the metal atoms in our STM images provides easy chemical identification, allowing precise determination of their coverage and structure. STM and x-ray photoelectron spectroscopy (XPS) measurements confirm the stability of the doped layer at high temperatures up to at least 400°C (*36*). We practically demonstrate the successful transfer of the doped layer onto a transmission electron microscopy (TEM) grid, further confirming the electrochemical stability of these dopants (*37*). Subsequent scanning TEM (STEM) with electron energy loss spectroscopy (EELS) provides direct and straightforward evidence of Co and Ni atom incorporation in Gr, confirming the robustness of the doped layer after transfer onto another support. Such materials are interesting as single active site catalysts to study reaction mechanisms or as sensors for selective gas adsorption at different metal atoms. Moreover, given the electrochemical stability of the incorporated metal atoms, these layers are highly promising as electrode materials in electrochemistry (*4*).

## RESULTS

### Synthesis of the Co-doped Gr layer

**Fig. 1.**
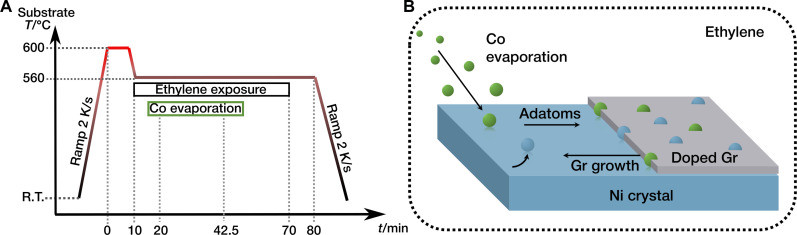
During-growth incorporation of Co and Ni into Gr on Ni(111) substrate. Details of the used recipe are sketched in the diagram of the substrate temperature versus time (**A**). The growth mechanism, schematically represented in (**B**), involves Co and Ni adatoms diffusing across the substrate surface before being incorporated in the growing edge of the Gr layer.

The idea of direct, during-growth incorporation of Co atoms was inspired by the previously reported mechanism for Gr doping with Ni atoms (*29*, *35*) and by the theoretical prediction of a catalytic action by Cu and Co during the growth process of a Gr layer (*37*). Details of the adopted procedure are provided in Fig. 1A. The last annealing step of the substrate cleaning protocol (see Materials and Methods) is used for Gr growth and Co incorporation, as follows. After keeping the sample for 10 min at 600°C under ultrahigh vacuum (UHV) conditions (<5 × 10^−10^ mbar), the temperature is decreased to 560°C, to grow Gr by 1-hour ethylene exposure at 5 × 10^−7^ mbar, until a full ML of Gr is formed. This procedure results in an epitaxial Gr layer aligned with the Ni(111) substrate. To favor single-atom doping, Co deposition is started after the first 10 min of ethylene exposure, i.e., when Gr nucleates and Co can get into contact with its growing edges, in analogy to the process previously reported for Ni incorporation (*29*). Co deposition continues for a total of 22.5 min, yielding a nominal coverage of 0.08 ML (see Materials and Methods). After stopping Co evaporation, Gr growth continues for a further 27.5 min in the ethylene atmosphere and an additional 10 min without ethylene before the final cooling to room temperature. To achieve a uniform distribution of the dopants, the timing of the Co deposition falls inside the main Gr formation window, i.e., when the layer rapidly grows after nucleation of its first flakes, while Co coverage and flux are kept low to minimize contamination of the Ni crystal and cluster formation. The incorporation efficiency of this process (percentage of finally incorporated Co atoms versus nominal Co coverage) is estimated to reach the value of about 2%. The non-incorporated fraction will mostly dissolve into the Ni bulk when reaching Gr-free areas (*38*), while impinging Co atoms on top of already-grown Gr flakes could intercalate at the Gr/Ni interface. Cluster formation on top of Gr can be excluded on the basis of our previous studies on the thermal stability of Co adatoms and clusters on Gr, which show that these are unstable on pristine Gr at room temperature (*39*) and, if anchored at Ni dopants, their stability increases up to 250°C (*40*).

Although we observe the incorporation of Co atoms in all experiments where Ni atom incorporation occurs, we present the recipe that optimizes the balance between the quality of the Gr layer and the Co dopant density. Several other variants of the process parameters were tested, including different growth temperatures and evaporation windows. However, these variations typically resulted in lower dopant concentrations at higher growth temperatures or poorer quality of the resulting layer at lower temperatures, particularly among rotational domains, thereby hampering the characterization of the layer.

Compared to other metal substrates, Ni promotes Gr growth already at rather low temperatures (*34*), limiting bulk dissolution of Co adatoms and alloy formation. Diffusion of adatoms across the surface is thus favored, increasing the probability of reaching a growing Gr edge. At the same time, also Ni adatoms diffusing on the surface can get trapped into the Gr mesh. Both processes are illustrated in Fig. 1B. It should be noted that the presence of Ni or Co single atoms at the Gr edge has a catalytic effect on the growth process of the Gr layer itself, favoring their incorporation into the mesh (*35*). Therefore, although in this study the method is applied to the specific case of Co doping, it should, in principle, be extendable to any type of TM dopant capable of catalyzing Gr growth (*41*), allowing for a variety of possible combinations that could unlock specific properties (*42*).

### Characterization and thermal stability

**Fig. 2.**
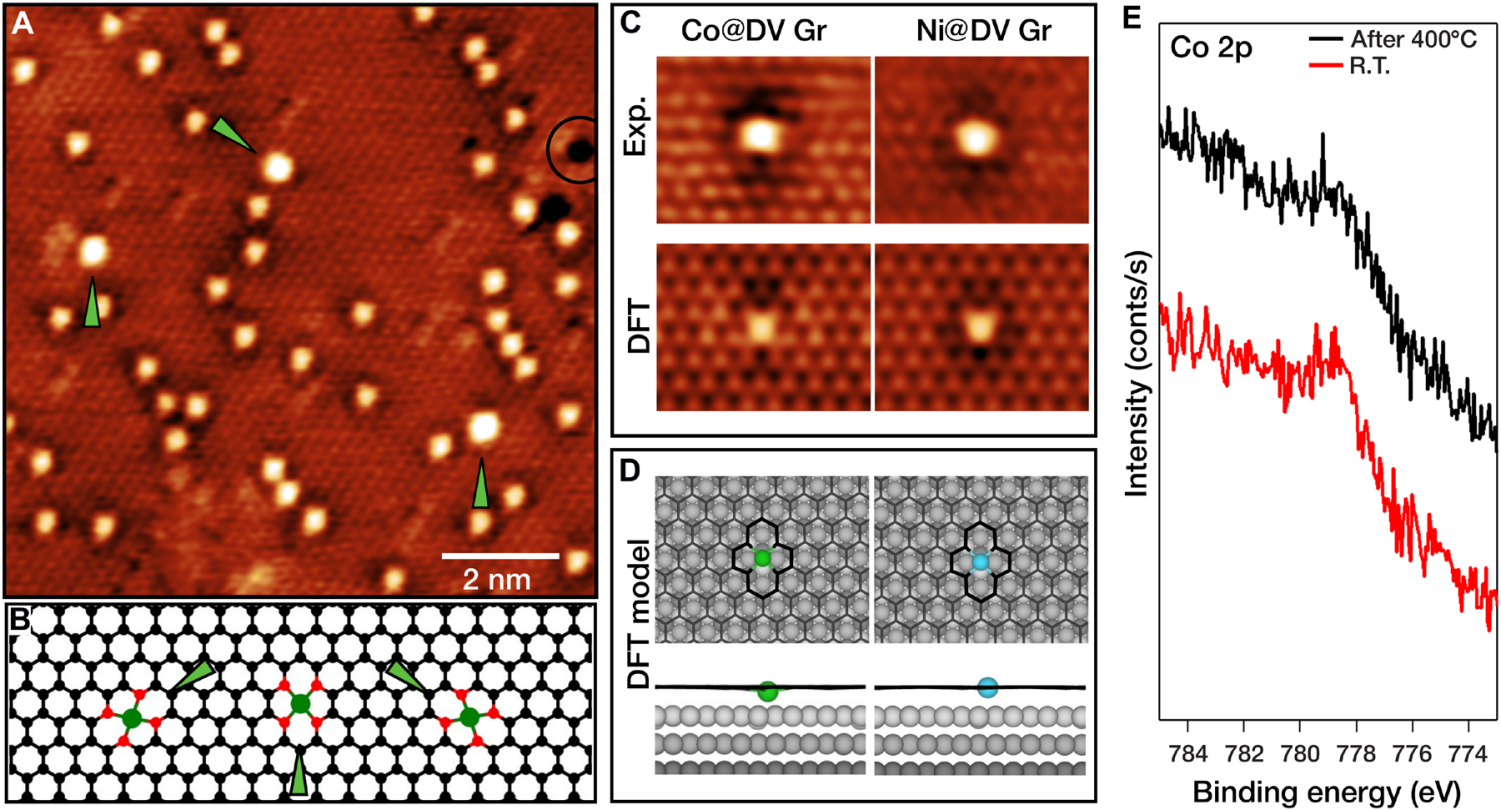
STM, DFT, and XPS characterization of the Co-Ni–doped Gr layer. The large-scale room temperature STM image in (**A**) shows the abundance of Ni versus Co dopants; the latter marked by green arrows. Three possible orientations of the Co dopants exist [see models in (**B**), where the C atoms coordinated to Co are colored in red]; two of them are visible in the image (A)—indicated by the arrow directions—and can be distinguished by their different appearance. Panel (**C**) shows a comparison between experimental and simulated STM images of Co and Ni atoms incorporated in the Gr mesh (*V*_b_ = −0.1 V, size 2 × 1.8 nm^2^). In (**D**), the related models in the top and side views are displayed [corresponding for Co to the configuration shown in (B) in the middle]. Panel (**E**) reports XPS spectra of the Co 2p core level at room temperature (R.T.) and after annealing at 400°C, respectively. Tunneling parameters: (A) *V*_b_ = −0.15 V, *I*_t_ = 1.0 nA, [(C), Co] *V*_b_ = −0.1 V, *I*_t_ = 1.7 nA, [(C), Ni] *V*_b_ = −0.2 V, *I*_t_ = 1.7 nA.

The Co-Ni–doped Gr layer obtained by the procedure described in the previous paragraph was characterized by VT-STM, DFT, and XPS. A representative STM image of the doped Gr at room temperature is shown in Fig. 2A. Bright protrusions corresponding to single metal atoms are randomly distributed within the Gr layer. Very few dark depressions are evident as well (marked in Fig. 2A by a black circle) and can be associated with single C vacancies (*29*). Closer inspection reveals that some features, highlighted by green arrows in Fig. 2A, appear brighter than the others, suggesting the presence of two different kinds of metal dopants, likely corresponding to Co and Ni. For an unambiguous identification, STM images of Co and Ni covalently bound to four C atoms inside a C double vacancy in a Gr layer (Co@DV Gr and Ni@DV Gr, respectively) were separately simulated by DFT calculations. Figure 2C compares high-resolution STM images (top) with simulated images (bottom), respectively, of Co@DV (left column) and Ni@DV (right column). The top and side views of the corresponding structural models are displayed in Fig. 2D. In the simulated STM images, the two dopants can be distinguished by the dark features of the surrounding Gr network, which serve as fingerprints: while the Ni dopant has a continuous dark shadow surrounding the pronounced bright metal center, Co displays alternating dark and bright features. Close comparison with the experimental images allows for the safe identification of the brighter features in the large-scale image in Fig. 2 as Co dopants. Furthermore, the orientation of the DV can be derived from the asymmetry of the dark features around the brighter Co atom, revealing that no preferential orientation appears in the STM images. As an example, two of the three possible orientations are visible in the STM image in Fig. 2 as modeled in Fig. 2B. It should be noted that the brightness of the central features strongly depends on the bias voltage: At −0.1 V, the calculated STM images do not exhibit the difference in brightness observed in the experimental STM image in Fig. 2A, acquired at −0.15 V. An example of the same area measured at −0.1 and −0.2 V is presented in fig. S1, demonstrating how the appearance of the different features is strongly affected by the bias. For this reason, we used different bias voltages to highlight different aspects of the structure while imaging.

Having identified the nature of the doping species, we can estimate their coverages from STM images, obtaining an abundance of 0.07% Co and 0.9% Ni with respect to C atoms in the Gr layer, corresponding to 0.025 Co atoms nm^−2^ and 0.33 Ni atoms nm^−2^, respectively. The fact that across the whole sample, Co and Ni appear intermixed and in the same structural configuration, even if their concentrations are quite different, supports the assumption that the same incorporation mechanism holds. Three important aspects of our growth method should be highlighted here. First, the Co concentration in the layer can be easily upscaled by increasing the incident flux (most likely leading also to an increased amount of bulk dissolved Co): as an example, using the same recipe with a 3× metal flux resulted in a 3× Co coverage increase (see Table 1, where each value was estimated from at least 10 frames). Second, the method can be successfully applied also to other Ni substrates [see fig. S2 for the Ni(100) case], suggesting its possible extension to more applicative cases, e.g., polycrystalline Ni foils. Last, the fact that Co is incorporated as a heteroatom alongside the naturally diffusing Ni adatoms suggests that this procedure is extendable to other heteroatoms that similarly interact with Gr, i.e., catalyzing Gr growth, especially at the growing edge (*41*).

**Table 1.** Determined dopant concentrations. Co and Ni concentrations [atoms (at.) per square nanometer and with respect to (w.r.t.) C atoms in the Gr layer] using high and low Co flux during the preparation (values in atoms per square nanometer and relative abundance in the Gr layer).

	Co (at. nm^−2^)	Co w.r.t. Gr	Ni (at. nm^−2^)	Ni w.r.t. Gr
Low Co flux (0.003 ML min^−1^)	0.025	0.07%	0.33	0.9%
High Co flux (0.009 ML min^−1^)	0.080	0.22%	0.33	0.9%

Our synthesis method allows TM incorporation to be obtained in a single step and leads to a high-quality doped Gr layer within 1 hour and 20 min, constituting a notable improvement with respect to the alternative methods reported so far, including the dopant beam dragging insertion (*30*) or vacancy formation upon directed damaging of the grown layer (*26*). Moreover, already for the low flux case, the density of Co atoms we obtain is the highest reported so far for any TM dopant, apart from Ni. For instance, the reported densities for the ion implantation of Mn (*28*) or the two-step implantation method of Au (*25*) atoms are 0.017 and 0.0017 atoms nm^−2^, respectively.

It has previously been reported that the stability of metal dopants inside Gr strongly relies on porphyrin-like N defects (*6*, *43*). However, theory predicts comparable stability in N-free Gr (*36*). Moreover, the thermal stability of Gr on Ni single crystals is influenced by the crystal’s history, including factors like subsurface C contamination. Annealing the layer after cooling down to room temperature may alter conditions, potentially affecting its ability to sustain heating cycles. To address this issue experimentally, we investigated the temperature stability of the incorporated Co and Ni atoms by high-temperature STM and XPS measurements after cooling down to room temperature. STM images acquired upon subsequent annealing at 400°C show that the dopants are still visible and distinguishable, with no apparent structural changes, as reported in fig. S3, indicating that they are stable at least up to this temperature. Furthermore, no changes were observed after cooling to room temperature.

This observation is corroborated also by XPS: The Co 2p spectrum, displayed in Fig. 2E, confirms the presence of Co through a broad peak at 778.6 eV, which, apart from intensity reduction, does not change substantially its shape after annealing at 400°C. This signal reduction could be attributed to further dissolution of non-incorporated Co atoms into the subsurface, at depth beyond the detection limit. Moreover, the valence band spectrum—which is highly sensitive to doping—shows for the Co-Ni–doped Gr a shift of about 0.3 eV toward a more n-type character of the Gr π states with respect to the Ni-doped Gr, as presented in fig. S4. This experimental observation is in line with the computational finding that TM atoms trapped in Gr are positively charged (Co > Ni; see table S1), transferring electrons to the Gr layer (Co > Ni; see table S1). Moreover, for Co-Ni–doped Gr, the total number of trapped metal atoms (Co and Ni) is expected to be higher than for Ni-doped Gr. Consequently, the n-doped character is expected to be more pronounced, due to a higher concentration of donor dopants.

### STEM and EELS characterization

As a final step of our investigation, we isolated the doped Gr layer from the Ni substrate, to characterize it by STEM, with a twofold aim: on the one hand, to obtain an independent assessment of their chemical nature; on the other hand, to test the possibility of transferring the two-dimensional network to a different substrate without losing the doping atoms, as expected since the M─C bonding should be more stable than the M-Ni substrate (*36*). Moreover, potential applications can be inferred from the demonstrated transferability and stability in electrochemical and ambient environments. Mass production of Gr via CVD (*44*, *45*) typically involves four main steps: growth on a metal substrate, removal of the substrate, transfer onto a desired support, and subsequent functionalization of the Gr layer. In this framework, the protocol that we propose offers substantial advantages, as it allows for substrate preservation and primary functionalization, resulting in well-defined and high-quality layers, thus eliminating the need for an additional functionalization step. Furthermore, by preserving the initial substrate, our method is potentially upscalable, thus avoiding the costs associated with substrate dissolution for detachment and enhancing the efficiency of CVD in large-scale production (*46*, *47*).

**Fig. 3.**
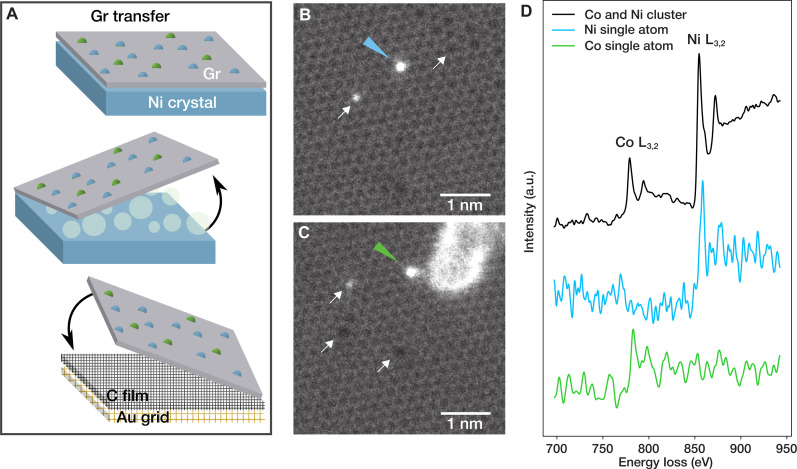
Substitutional Ni and Co dopants in Gr characterized by STEM and EELS after transferring the layer onto a TEM grid. The transfer process is schematically displayed in (**A**), illustrating the initial stage on the Ni substrate, the delamination through bubbling, and the transfer onto the Quantifoil Au grid stack. STEM–medium angle annular dark-field (MAADF) images of two different regions of ML Gr, containing respectively one substitutional Ni atom [(**B**), blue arrow] and one substitutional Co atom [(**C**), green arrow]. As seen in the images, the Gr lattice also contains other defects (white arrows), such as vacancies and lighter impurities, mainly Si. (**D**) EELS point spectra for single Ni and Co atoms are shown in blue and green, respectively. The spectra were scaled to have a similar magnitude for the peaks. The black spectrum was recorded as a reference from a metal cluster containing both Co and Ni atoms (see Supplementary Materials for the corresponding STEM-MAADF image).

First, the doped Gr layer, prepared in the STM chamber according to the recipe described above, was transferred onto TEM grids using the “bubbling” method (*48*), as described in Materials and Methods. STEM measurements reveal a high Gr coverage on the Quantifoil grids, demonstrating the appropriateness of the transfer process and the stability of the doped Gr layers during the electrochemical treatment. After transfer, the Gr surface was largely covered with hydrocarbon contamination, preventing its characterization. To remove contamination, the samples were exposed to laser irradiation (10 ms at 5 mW) inside the microscope column (*49*, *50*), resulting in large (hundreds of nanometer) atomically clean areas. Some of these areas appeared as nearly defect-free Gr lattice, while others contained a notable number of impurities and other defects. Direct identification of the impurity atoms was obtained by acquiring EEL spectra directly on the metal dopants. Figure 3 (B and C) show two examples of impurities (highlighted by blue and green arrows) within the Gr lattice, whose corresponding single-atom EEL spectra are reported in Fig. 3D. A reference spectrum, measured from a metal cluster containing Co and Ni, is also displayed in Fig. 3D (corresponding image in fig. S5). The clear presence in the EEL spectra from the impurities of the peaks that correspond to L_3,2_ edges of Co (779 and 794 eV) and Ni (855 and 872 eV) unambiguously confirm their attribution to Co and Ni single-atom dopants, respectively. The dopants show a remarkably different behavior under electron irradiation. While Ni appears to be very stable, Co dopants readily escape from the lattice under the electron beam, making EELS characterization very challenging. Figure 3 (B and C) also show the presence of other defects, indicated by white arrows, such as structural defects and dimmer impurity atoms, most probably Si. We note that laser cleaning and high-energy electron irradiation are known to eject some metal dopants (*25*), leaving in the Gr lattice vacancy defects, that can be subsequently filled by C or Si atoms originating from the sample support or the environment (*51*).

## DISCUSSION

The presented method efficiently incorporates Co and Ni single atoms into a growing Gr layer on a Ni(111) substrate. The synthesis extends the previously reported during-growth Ni atom incorporation mechanism occurring on Ni substrates at moderate temperatures. Structural characterization of the doped layer was performed by variable temperature STM measurements, revealing a high-quality Gr layer, codoped with Ni and Co atoms in C DVs, with the highest dopant concentrations up to date. STM distinguishes Co and Ni atoms in the Gr mesh based on their distinct electronic structures, aligned with simulated DFT images. Thermal stability of the dopants in the Gr layer up to at least 400°C was demonstrated by STM and XPS measurements. The doped Gr layer was exposed to the ambient atmosphere and electrochemically delaminated. After transfer onto a TEM grid, additional investigation by STEM and EELS provides direct and straightforward evidence of the presence of the Co and Ni dopants in the freestanding phase, standing even the aggressive transfer treatment.

The fact that the Gr layer grows at such low temperatures on Ni not only offers an efficient synthesis procedure in terms of conditions and resources but is also the key factor for the successful incorporation of Co into Gr. We expect that this doping method can be used with any TM adatom capable of catalyzing Gr growth, opening the possibility for versatile metal incorporation toward tailored materials for a variety of specific applications. Furthermore, the characteristics of the proposed method, based on simple evaporation during CVD growth, point to its intrinsic scalability. The fact that the concentration is still relatively low (<1%) is not necessarily a drawback. The dopants are uniformly distributed in a specific structure, isolated from neighboring centers. Too high concentrations could lead to predominant additional configurations with too close neighboring atoms, disturbing the single active site character, seen by chain-like Ni dopant arrangements at lower growth temperatures in the supporting information of related work (*29*). In their STM images (see fig. S1), the Ni dopant density increases with lower growth temperatures (<520°C), and chain-like arrangements of the Ni dopants appear, which could alter the reactivity. We further exclude the ability to grow long-range periodic arrangements with this method. However, this system is highly promising for specific relevant applications where such an order is not required. The transferability of the layer paves the way for potential applications in spintronics, electrical devices, and electrode materials for single-atom catalysis or heterogeneous catalysis. This highly efficient and simple method for incorporating metal atoms into Gr is therefore expected to have a notable impact on nanoscience and technology, potentially paving the way for the commercial use of functionalized Gr by CVD, given its advantages in terms of purity, scalability, controlled doping, and enhanced catalytic properties.

## MATERIALS AND METHODS

### Experimental design

A synthesis route to uniformly dope Gr with TMs is the objective of this study. The doped Gr layer was characterized at atomic resolution by STM, corroborated by DFT calculations. The potential of the system was assessed upon air exposure, electrochemical delamination, and transfer onto another support; in this case, the TEM grid to demonstrate the persistence of the functional metal dopants by STEM/EELS experiments.

### Synthesis of the Co- and Ni-doped layer

Synthesis of the Co- and Ni-doped Gr layer was conducted in a UHV preparation chamber (base pressure, 1 × 10^−10^ mbar) equipped with standard tools for sample preparation, such as an Ar^+^ sputter gun, gas line with ethylene, and manipulator with a heating stage. Co was evaporated by means of an electron beam evaporator from Omicron with flux control. The parameters used were 0.003 ML min^−1^ flux corresponding to a nominal coverage of 0.07% with respect to Gr and for the high flux 0.009 ML min^−1^ (0.22%), determined by STM images of Co on the bare Ni surface. Low-energy electron diffraction was used to confirm the orientation of Gr. The detailed during-growth synthesis for Co incorporation in Gr is described in Results. To clean the Ni substrate, three subsequent cycles were conducted, including sputtering at 2 × 10^−6^ mbar Ar^+^ pressure, 2 kV for 10 min, and annealing at 600°C for 10 min with 2 K s^−1^ heating ramps.

### STM experiments

STM measurements in the constant current mode were performed with an Omicron VT-STM operated by an R9plus controller (RHK Technology). For the measurements of the temperature stability of Co dopants, the sample was annealed directly in the STM stage at about 1 K s^−1^ and stabilized at the desired temperature for about 5 min before proceeding with the measurements. The STM images were analyzed and processed using standard operations by means of the Gwyddion software (*52*).

### XPS/UPS experiments

XPS and ultraviolet photoelectron spectroscopy (UPS) measurements were performed ex situ, after transferring the sample by means of a high-vacuum suitcase, at the INSPECT laboratory at CNR-IOM, located in Basovizza, Trieste. The sample was measured as transferred and after annealing at 400°C to desorb any possible contaminant and, at the same time, verify the stability of the Co-Ni–doped layer. Photoemission spectra were acquired at normal emission with a PSP analyzer 120 mm, a 5 Channeltron detector, and a Mg Kα x-ray source without a monochromator. The base pressure of the chamber was 5 × 10^−10^ mbar, while during measurement, the pressure was in the 10^−9^ mbar range. The valence band spectra were acquired with the same analyzer with a non-monochromated He lamp.

### Transfer onto the TEM grid

Before the transfer process onto the TEM grid, the sample was subjected to CO intercalation inside a high-pressure cell connected to the UHV chamber at 2 torr for ~2 hours, lifting the layer into a quasi-freestanding phase (*53*, *54*) to facilitate the transfer. The delamination of the layer from the single crystal was conducted through an electrochemical transfer process (so-called the “bubbling” method), for preserving the substrate material (*48*). Specifically, the Gr/CO/Ni(111) sample was spin coated with polymethyl methacrylate (PMMA) at 3000 rpm for 1 min and transferred to Quantifoil holey carbon TEM grids (Au 200 mesh, R 2/2) by electrochemical delamination. Here, an aqueous 1 M NaOH solution was used as an electrolyte, while the Ni substrate and a Pt wire acted as cathode and anode, respectively. Gas bubbles formed at the interface of the Ni substrate and Gr during this process exert enough force to separate the layer. The current varied between 0.2 and 1.0 A. After complete delamination, the PMMA/Gr stack was cleaned in deionized water for 1 hour, fished out with the TEM grid, and dried on a heating plate at 75°C for 2 hours before removing the PMMA in consecutive acetone (70°C) and isopropanol baths.

### STEM/EELS experiments

After the transfer, the Co- and Ni-doped Gr layer was characterized at University of Vienna with an aberration-corrected Nion UltraSTEM 100 at 60 kV. The angular range of the medium angle annular dark-field detector was 60 to 200 mrad, and the convergence semiangle was ~30 mrad. EELS was carried out with a Gatan PEELS 666 spectrometer with an Andor iXon 897 electron-multiplying charge-coupled device camera. The energy dispersion was 0.3 eV pixel^−1^.

### Theory

DFT calculations were performed using the plane wave–based Quantum ESPRESSO package (QE) (*55*, *56*). Ultrasoft pseudopotentials (*57*, *58*) were adopted to describe the electron-ion interaction with Ni (4s, 3d), Co (4s, 3d), and C (2s, 2p), treated as valence electrons. Energy cutoffs of 47 and 326 rydberg (Ry) for kinetic energy and charge density expansion, respectively, were adopted for all calculations. The convergence criterion of 0.026 eV/Å for the forces was used during geometry optimization, and the convergence criterion for the total energy was set at 10^−6^ Ry. To properly consider the dispersion interactions, the van der Waals density functional vdW-DF2^C09x^ was used (*59*, *60*), as implemented in the QE code. This functional was proven to give an accurate description of the adsorption energies and distances of Gr on metal surfaces (*60*) and has also been successfully applied to describe other two-dimensional/metal interfaces (*61*). Spin polarization was always included. Atomic charges were calculated using the Bader scheme (*62*).

For the simulation of M@DV Gr interfaces (where M = Co or Ni), a 6 × 6 supercell was used, with a total of 108 Ni atoms in the Ni(111) substrate, 70 C atoms in the Gr layer, and 1 Co or Ni atom in the Gr DV. A Monkhorst-Pack (*63*) k-points mesh of 3 × 3 × 1 was used for the geometry relaxation. The Ni(111) surface was modeled by a three-layer slab with a bottom layer fixed to the bulk positions during the geometry relaxation to mimic a semi-infinite solid. To avoid interactions between adjacent periodic images, a vacuum of about 24 Å in the direction perpendicular to the surface together with a dipole correction was used.

STM simulations were performed using the Tersoff-Hamann approach (*64*), and constant-current and bias voltage values for the STM simulations were chosen to match the experimental values. Ball-and-stick models and STM images were rendered with VESTA (*65*) and Gwyddion (*52*) software, respectively.
