# Supplementary material for: Scalable bottom-up synthesis of Co-Ni–doped graphene
Source: Sci Adv. 2024 Nov 8;10(45):eado8956. doi: 10.1126/sciadv.ado8956 (PMC11546814; doi:10.1126/sciadv.ado8956)
Supplement: Supplementary file 1 — Figs. S1 to S5 Table S1 [file sciadv.ado8956_sm.pdf]

Science Advances
AAAS

Supplementary Materials for

Scalable bottom-up synthesis of Co-Ni–doped graphene

Valeria Chesnyak *et al.*

Corresponding author: Cristina Africh, africh@iom.cnr.it

Sci. Adv. **10**, eado8956 (2024)
DOI: 10.1126/sciadv.ado8956

This PDF file includes:

Figs. S1 to S5
Table S1

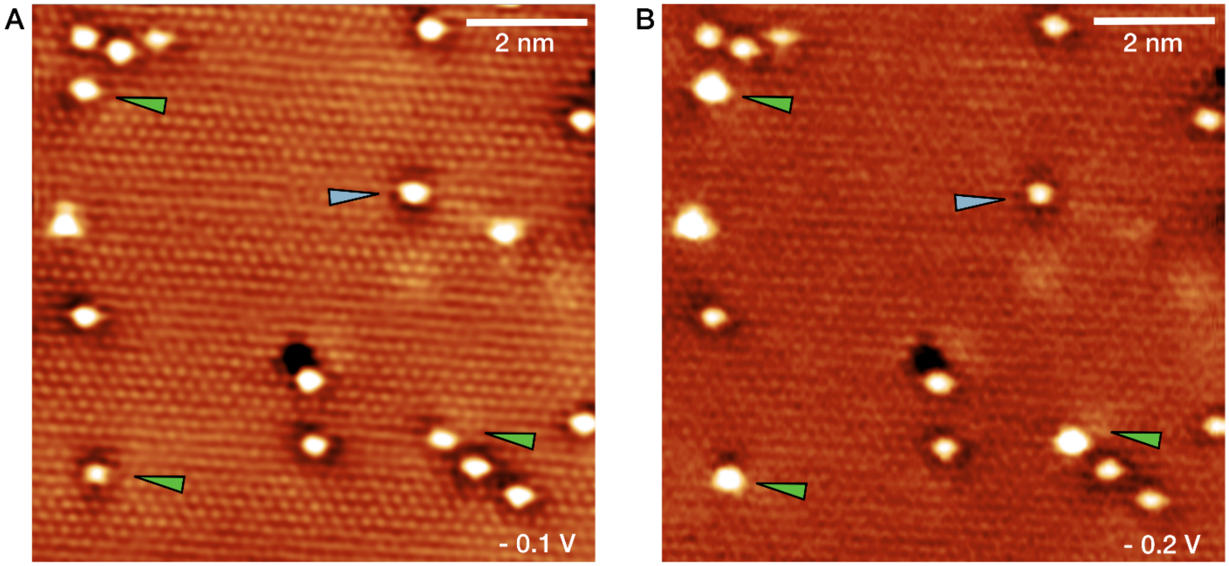

fig. S1.

Comparison of two STM images of same area acquired at different bias voltage. Co/Ni atoms are indicated by green/blue arrows, respectively. Tunneling parameters: (**A**) $V_b = -0.1$ V, $I_t = 1.7$ nA, (**B**) $V_b = -0.2$ V, $I_t = 1.7$ nA.

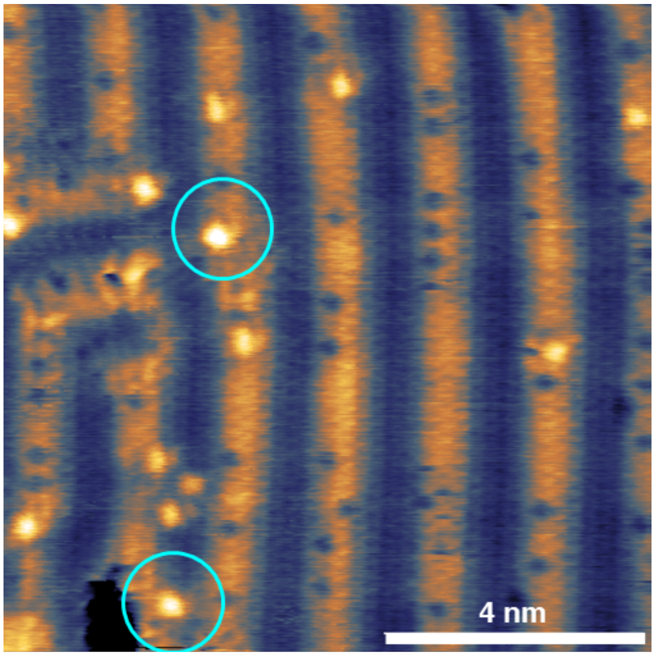

fig. S2.

STM image showing metal dopants in Gr/Ni(100), encircled in light blue. Direct comparison with DFT simulated images and assignment of the defects to Co and Ni is difficult due to the presence of the moiré structure. Tunneling parameters: $V_b = 0.2$ V, $I_t = 2.7$ nA.

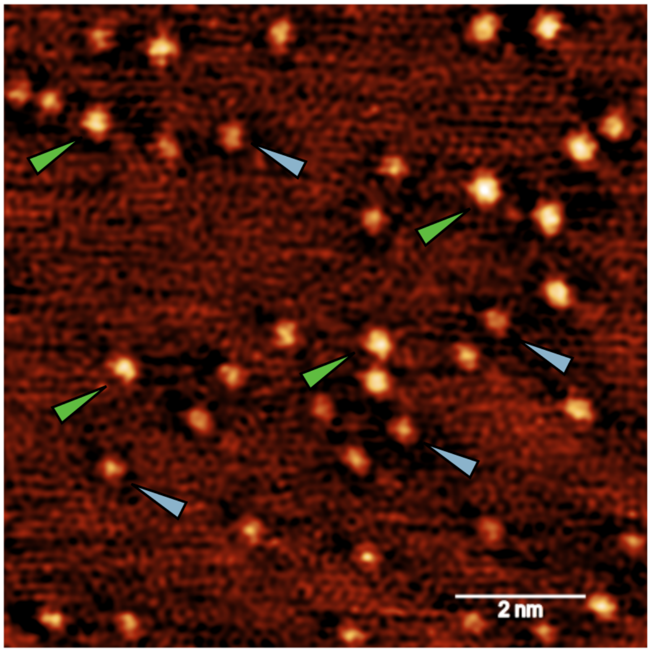

fig. S3.

STM image taken at 400 °C showing the persistence of both Co and Ni dopants in the Gr network, as highlighted by the green and blue arrows, respectively. Tunneling parameters: V_b = -0.2 V, I_t = 0.8 nA, size 10x10 nm^2.

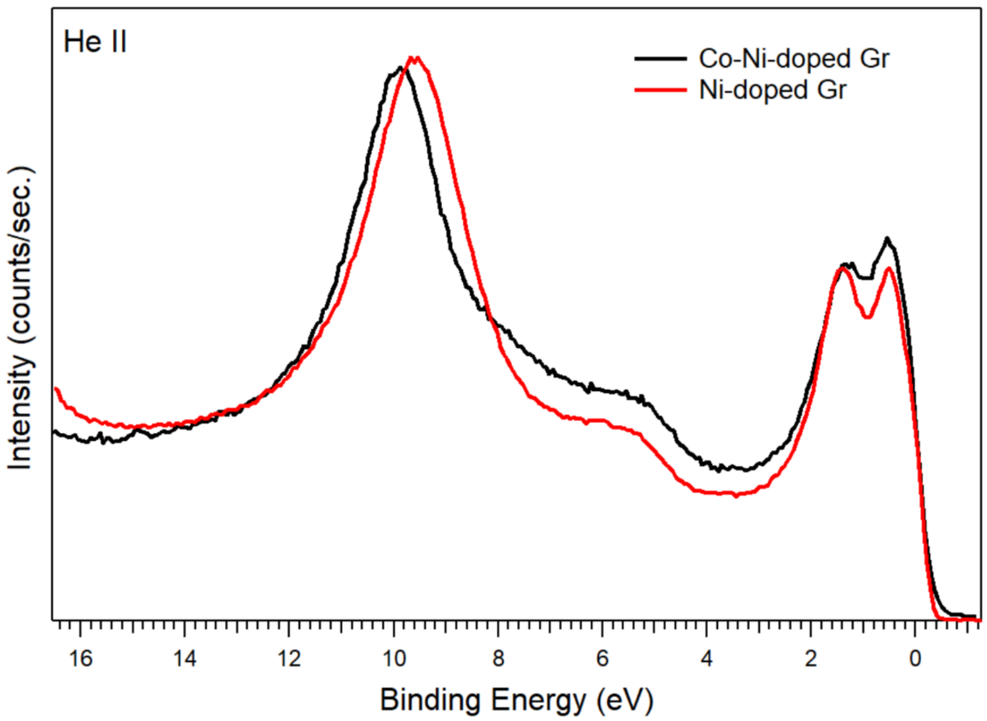

fig. S4.

UPS investigation of Ni-doped and Co- and Ni-doped Gr. Comparison of UPS of the valence band for Co-Ni-doped Gr (after annealing at 400 °C, in black) and Ni-doped Gr (in red) showing a shift of 0.3 eV of the Gr π band around 10.3 eV.

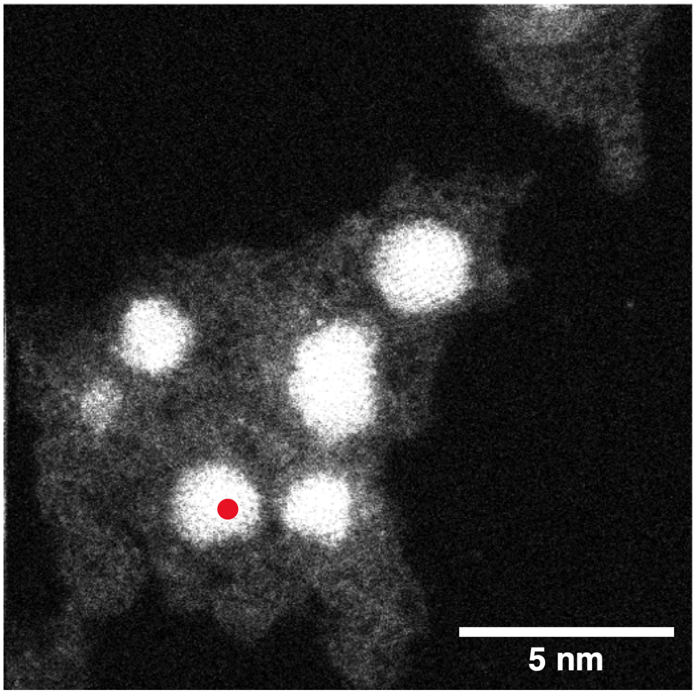

fig. S5.

STEM-MAADF image of metal clusters consisting of Co and Ni. The dark area corresponds to clean Gr, the light-gray area is carbon-based contamination, and the bright features are metal clusters. The position of the probe for acquiring the EEL spectrum shown in Fig. 3 in the main article is indicated by a red point.

Table S1.

Changes in atomic charges (in e^-/supercell) with respect to the isolated atom, calculated through Bader analysis for Gr/Ni, DV Gr, Ni@DV Gr, and Co@DV Gr systems. Negative (positive) values mean electron charge accumulation (depletion). The second, third, and fourth column report the charge variation on a subgroup of atoms of each system: the Gr layer only, the Ni(111) substrate only, and the Ni/Co adatoms trapped in Gr.

Atomic charge (e^-)	**Gr**	**Ni (111)**	**Co/Ni**
**Gr/Ni**	-4.62	+4.62	-
**DV Gr**	-4.46	+4.46	-
**Ni@DV Gr**	-4.88	+4.18	+0.70
**Co@DV Gr**	-5.19	+4.38	+0.80
